# The *Leishmania donovani* histidine acid ecto-phosphatase *Ld*MAcP: insight into its structure and function

**DOI:** 10.1042/BJ20141371

**Published:** 2015-04-17

**Authors:** Amalia Papadaki, Anastasia S. Politou, Despina Smirlis, Maria P. Kotini, Konstadina Kourou, Thomais Papamarcaki, Haralabia Boleti

**Affiliations:** *Intracellular Parasitism Group, Department of Microbiology, Hellenic Pasteur Institute, Athens 11521, Greece; †Laboratory of Biological Chemistry, Medical School, University of Ioannina, Ioannina 45110, Greece; ‡Biomedical Research Division, Foundation for Research and Technology-Hellas, Institute of Molecular Biology and Biotechnology, Ioannina 45110, Greece; §Molecular Parasitology Laboratory, Department of Microbiology, Hellenic Pasteur Institute, Athens 11521, Greece; ║Light Microscopy Unit, Hellenic Pasteur Institute, Athens 11521, Greece

**Keywords:** ecto-enzyme, histidine acid phosphatase, *Leishmania* virulence, aa, amino acids, Ab, antibody, BLAST, Basic Local Alignment Search Tool, BW, black and white, Ct, cycle threshold value, EMBL, European Molecular Biology Laboratory, EndoF, endoglycosidase F, ER, endoplasmic reticulum, FL, fluorescence, FP, flagellar pocket, HAcP, histidine acid phosphatase, hiFBS, heat-inactivated FBS, hPAP, human prostatic acid phosphatase, IF, immunofluorescence, *Ld*MAcP, *Leishmania donovani* membrane acid phosphatase, *Ld*SAcP, *Leishmania donovani* secreted acid phosphatase, mAb, monoclonal antibody, mRFP, monomeric RFP, ORF, open reading frame, pAb, polyclonal antibody, Pi, inorganic phosphate, PI, propidium iodide, *p*NP, *p*-nitrophenolate, *p*NPP, *p*-nitrophenyl phosphate, qPCR, quantitative PCR, RPMI, Roswell Park Memorial Institute medium, RT, room temperature, SP, signal peptide, TM, transmembrane, wt, wild type

## Abstract

Acid ecto-phosphatase activity has been implicated in *Leishmania donovani* promastigote virulence. In the present study, we report data contributing to the molecular/structural and functional characterization of the *L. donovani Ld*MAcP (*L. donovani* membrane acid phosphatase), member of the histidine acid phosphatase (HAcP) family. *Ld*MAcP is membrane-anchored and shares high sequence identity with the major secreted *L. donovani* acid phosphatases (*Ld*SAcPs). Sequence comparison of the *Ld*MAcP orthologues in *Leishmania* sp. revealed strain polymorphism and species specificity for the *L. donovani* complex, responsible for visceral leishmaniasis (Khala azar), proposing thus a potential value of *Ld*MAcP as an epidemiological or diagnostic tool. The extracellular orientation of the *Ld*MAcP catalytic domain was confirmed in *L. donovani* promastigotes, wild-type (wt) and transgenic overexpressing a recombinant *Ld*MAcP–mRFP1 (monomeric RFP1) chimera, as well as in transiently transfected mammalian cells expressing r*Ld*MAcP–His. For the first time it is demonstrated in the present study that *Ld*MAcP confers tartrate resistant acid ecto-phosphatase activity in live *L. donovani* promastigotes. The latter confirmed the long sought molecular identity of at least one enzyme contributing to this activity. Interestingly, the *L. donovani* r*Ld*MAcP–mRFP1 promastigotes generated in this study, showed significantly higher infectivity and virulence indexes than control parasites in the infection of J774 mouse macrophages highlighting thereby a role for *Ld*MAcP in the parasite's virulence.

## Abstract 2

Acid ecto-phosphatase activity has been linked to *Leishmania donovani* virulence. In the present study, we confirm the molecular identity and characterize molecular and functional properties of an enzyme contributing to this activity, the *Ld*MAcP, an *L. donovani* specific membrane histidine acid phosphatase (HAcP).

## INTRODUCTION

Species of the parasitic protozoan genus *Leishmania* are the causative agents of a wide variety of human cutaneous and visceral diseases known as leishmaniases [1]. These organisms reside, throughout their digenetic life cycles, in hydrolytic environments i.e. in the alimentary tract of the sandfly vector host as extracellular flagellated promastigote forms and within the phagolysosomal system of macrophages in their mammalian hosts as obligate intracellular forms [2,3]. All physiological and biochemical interactions of the parasite with its host cell occur, at least temporarily, at the surface or across the parasite plasma membrane, which must be traversed by the organism's nutrients, as well as by its secreted and excreted metabolic products. In accordance with this, membrane bound ecto-enzymes with their active sites facing the extracellular medium, are anticipated to play a critical role in the survival and maintenance of the parasite within the infected host.

One class of such enzymes are ecto-phosphatases that hydrolyse extracellular phosphorylated substrates and release free inorganic phosphate (Pi). Their activities can be measured in live cells. Due to their extracellular active site, ecto-phosophatases may enable organisms to obtain necessary nutrients from organic phosphates in their environment [4]. Indeed, it has been shown that growth of certain trypanosomatid parasites depends strongly on the presence of Pi in the culture medium [5] and its replacement by β-glycerophosphate, a phosphatase substrate, results in parasite maximal growth. Additionally, Pi-starvation results in a significant increase in parasite ecto-phosphatase activity [6] that correlates with parasite proliferation [5].

Acid ecto-phosphatases that function at acidic pH are ubiquitous in Nature. Enhanced acid ecto-phosphatase activity in protozoan cells may be important for (i) adaptation in acidic environments, (ii) acquisition of nutrients from host cell phosphorylated substrates or (iii) survival [7,8] within phagocytes through inhibition of the respiratory burst [9]. Therefore, these enzymes could act as virulence factors. Acid ecto-phosphatase(s) isolated from the external membrane of *Leishmania donovani* promastigotes inhibit production of superoxide anions by human neutrophils [10], suggesting that parasites with greater ecto-phosphatase activity would be more resistant to oxidative stress within the phagolysosome. Indeed, ecto-phosphatase activity from *L. donovani* and *Trypanosoma cruzi* promastigotes seems to be more resistant to H_2_O_2_ than ecto-phosphatase from non-pathogenic protozoans [11,12]. In addition, sensing of reactive oxygen species (ROS) is likely to be important for the adaptation and survival of trypanosomatids or other protozoan parasites within the environment of different hosts [9,12–14]. Phosphatases that possess a critical cysteine residue in their active site can mediate redox signalling through reversible oxidative inactivation [15,16].

The identification and partial characterization of an acid ecto-phosphatase activity localized on surface membranes of *L. donovani* that is responsible for the severe and potentially fatal visceral leishmaniasis was first reported by Gottlieb and Dwyer [4]. Subsequent studies have partially characterized the biochemical properties of this extracellular acid phosphatase activity and provided evidence for its relationship with the degree of promastigote infectivity/virulence [17–19]. The molecular identity of the surface membrane *L. donovani* acid phosphatase(s) remained elusive until the isolation of a gene which encodes a putative MAcP [membrane-bound histidine acid phosphatase (HAcP)] designated as *Ld*MAcP (*L. donovani* membrane acid phosphatase) [20]. Interestingly, this gene was found to be highly homologous to the *Ld*SAcP1 (secreted *Leishmania donovani* acid phosphatase) and *Ld*SAcP2 secreted HAcPs from *L. donovani* [21].

The superfamily of HAcP is a large and functionally diverse group of proteins, divided into two branches, which share the conserved motif RHGXRXP in their catalytic core domain. The histidine residue in this structural motif serves as a nucleophile in the formation of a covalent phosphohistidine intermediate [22]. Human representatives of both branches are of considerable medical interest [23] and several phosphatases, notably phytases, have current or potential applications in agriculture [24]. Other members of the HAcPs superfamily exist in various pathogenic microorganisms [25] and their inhibition might have therapeutic value [22].

In the current study, we aimed to elucidate the localization and structural/functional properties of *Ld*MAcP by studying the endogenous enzyme and the recombinant r*Ld*MAcP–mRFP1 and r*Ld*MAcP–His chimeras in *L. donovani* promastigotes and mammalian cells. The experimental evidence presented in the present study shows that *Ld*MAcP is specific for the *L. donovani* complex and is one, if not the only, enzyme contributing to the *L. donovani* acid-ecto-phosphatase activity. It also provides new insight into the *Ld*MAcP structure and its possible function in *L. donovani* virulence.

## EXPERIMENTAL

### Reagents and antibodies

All chemicals used, unless otherwise stated, were of analytical grade and purchased from Sigma or Applichem. Enzymes and DNA molecular mass standards were from Roche (New England Biolabs) and protein molecular mass standards from Amersham Biosciences. The mouse monoclonal 6× histidine epitope-tag antibody (Ab) was from Acris Antibodies, the anti-calnexin rabbit polyclonal Ab (pAb) and the anti-α-tubulin mouse monoclonal Ab (mAb) were from Sigma whereas the anti-BiP (endoplasmic reticulum binding protein GRP78) rabbit pAb was a kind gift from Dr J. D. Bangs, U. at Buffalo (SUNY). All Fluorochrome-conjugated secondary Abs (Alexa Fluor® 546, Alexa Fluor® 488 and Alexa Fluor® 633-conjugated to anti-rabbit or anti-mouse Abs) were from Molecular Probes.

### Cell culture

The murine monocytic J774 (A.T.C.C.) and the HeLa mammalian [26] cell lines were cultured in high glucose RPMI (Roswell Park Memorial Institute medium) 1640 and Dulbecco MEM (modified Eagle's medium; Gibco) respectively, containing 10% (v/v) heat-inactivated FBS (hiFBS; Gibco), 1 unit/ml penicillin and 0.1 mg/ml streptomycin. Both cell lines were maintained at 37***°***C in a 5% (v/v) CO_2_-humidified atmosphere.

*L. donovani* (strain LG13, MHOM/ET/0000/HUSSEN; [27]), *Leishmania major* Friedlin (reference strain, MHOM/IL/80/Friedlin, zymodeme MON-103; [28]) and *L. infantum* (strain GH12 [29]) promastigotes were at 25**°**C as described previously [30]. *Leishmania tarentolae* promastigotes (strain Parrot, Jena Biosciences) were cultured at 25***°***C in Brain Heart Infusion (BHI) medium supplemented with haemin (Jena Biosciences) at a final concentration of 0.25% (w/v), 10 μM L-biopterin (Cayman), 1 unit/ml penicillin and 0.1 mg/ml streptomycin.

### DNA constructs and cell transfections

The plasmid pRSET-b–mRFP1 (Invitrogen, MTA-Y.Tsien lab) was used as a template for the PCR amplification of the mRFP1 gene which was cloned into the *Bgl*II/*Xho*I sites of the *Leishmania* expression vector pLexsy-sat (pF4X1.4sat, Jena Biosciences) to produce the pLexsy-sat–mRFP1 plasmid.

The gene encoding *Ld*MAcP [1–315 amino acids (aa), GenBank] was amplified by PCR from genomic *L. donovani* DNA (strain LG13) and inserted into (i) the *Bgl*II site of the pLexsy-sat–mRFP1 plasmid to create the pLexsy–r*Ld*MAcP–mRFP1 plasmid and (ii) the *Bgl*II/*Xho*I sites of the pTriEx1.1 vector (Invitrogen), in frame with the C-terminal histidine-tag to produce the pTriEx1.1–*Ld*MAcP-His plasmid, used for transient transfection of mammalian cells Finally, a smaller fragment of the *Ld*MAcP gene (coding for aa 1–274) was cloned into the pTriEx1.1 vector to produce the pTriEx1.1–*Ld*MAcPsol–His plasmid for expression in *Escherichia coli* BL21 strain. Two positive clones were selected and sequenced in each case (VBC-Biotech). All primer pairs for the PCR reactions are listed in Supplementary Table S1. Genomic DNA from *L. major* Friedlin and *L. tarentolae* parrot was used for the PCR amplification of the LmjF.36.6460 and LtaP34.3910 genes.

*L. donovani* parasites transfected with supercoiled circular pLexsy-sat [15] and pLexsy-sat–r*Ld*MAcP–mRFP1 plasmids (episomal expression), were generated as previously described [30]. Selection of transgenic promastigotes was performed in RPMI 1640 [20% (v/v) hiFBS] containing 100 μg/ml Nourseothricin (Jena Biosciences).

Transfections of mammalian cells were performed after plating at a density of ∼1×10^6^/well in six-well plates. The pTriEx1.1–*Ld*MAcP–His and the pDisplay-mcherry (provided by I. Tardieux, Institut Cochin, Paris, France) plasmids were transfected using Lipofectamin Plus®, as described by the manufacturers (Invitrogen). Transfected cells were analysed by immunofluorescence (IF) and/or for acid ecto-phosphatase activity at 24 h post transfection.

### qPCR

Reverse transcription was carried out with total RNA from wt *L. donovani* (strain LG13) and *L. donovani* r*Ld*MAcP–mRFP1 stationary phase promastigotes, extracted by the hot acid phenol method [31]. A volume of 5 μl of diluted cDNA sample was used as template in 20 μl of SYBR Green (KAPA Biosystems) based quantitative PCR (qPCR) reactions performed on the Exicycler 96 (BioNEER). The qPCR protocol used was: 10 s at 94°C, 35 cycles comprising 45 s at 94°C, 30 s at 50°C, 20 s at 72°C and a final step of 10 min at 72°C. Expression of the *Ld*MAcP, mRFP1 and *GADPH* (glyceraldehyde-3-phosphate dehydrogenase; LinJ.36.2480) genes was analysed using the primers listed in Supplementary Table S1. Analysis of the qPCR data from two biological samples per experiment assayed in triplicates was performed by the comparative C_T_ method [32]. The cycle threshold value (Ct) for each sample, resulted from the average values of the duplicate samples. Expression of the genes of interest was estimated by the following equation:

2^−ΔΔ^Ct=[(Ct gene of interest − Ct internal control) r*Ld*MAcP–mRFP1 − (Ct gene of interest − Ct internal control) wt]

### Production of recombinant proteins and generation of antibodies

The recombinant mRFP1–His and *Ld*MAcPsol–His proteins (pRSET-b–mRFP1 and pTriEx1.1–*Ld*MAcPsol plasmids) expressed in *E. coli* BL21 cells were used to produce the anti-RFP and anti-*Ld*MAcP pAbs, as previously described [30]. The recombinant proteins were purified from bacterial lysates by metal-affinity chromatography (Qiagen Ni–NTA Superflow resin) and were subsequently injected into New Zealand white rabbits and BALB/c mice to raise pAbs according to published protocols [30,33]. All experimental procedures were approved by the Institutional Animal Bioethics Committee following the EU Directive 2010/63 and the National Law 2013/56. The anti-RFP pAb was affinity purified over cyanogen bromide (CNBr)-activated Sepharose 4B matrix (Pharmacia) to which the respective antigen was covalently attached. The mouse anti-LG13 pAb was raised after immunization of BALB/c mice with total *L. donovani* (strain LG13) extracts.

Mouse mAbs were generated against the E-F-A-R-S-R-Y-N-D-L-S-L (Glu^73^-Leu^84^) *Ld*MAcP peptide sequence according to protocols developed by the European Molecular Biology Laboratory (EMBL) monoclonal facility and carried out by Paratopes Ltd. Culture supernatants from the hybridoma cultures were used at 1:20 dilution for Western blot and at 1:2 dilutions for IF.

### Immunofluorescence

*Leishmania* promastigotes were fixed, allowed to attach on poly-L-lysine coated coverslips and labelled with primary and secondary Abs, as previously described [15,34]. The parasite DNA was stained [10 min, room temperature (RT)] with 10 μg/ml propidium iodide (PI) in PBS containing 100 μg/ml RNase. Coverslips were mounted with Mowiol 4-88 [10% (w/v) Mowiol-Calbiochem, 25% (v/v) glycerol, 100 mM Tris/HCl, pH 8.5] on microscope slides, sealed with nail polish and stored at 4°C. HeLa cells expressing the r*Ld*MAcP–His and pDisplay-mcherry chimeras or macrophages infected with *Leishmania* promastigotes were fixed with paraformaldehyde [4% (w/v) in PBS], stained with primary and secondary Abs or phalloidin-Alexa-546 and mounted as described above. For detection of surface extracellular epitopes, live HeLa cells expressing the r*Ld*MAcP–His or *Leishmania* promastigotes were washed with PBS and resuspended in PBS (1% w/v BSA) buffer containing the mouse anti-*Ld*MAcP pAb (dilution 1:50) for 20 min at 4°C. Subsequently, fixation (for the *Leishmania* parasites) and incubation with secondary Abs was performed as described above. Microscopic analysis of the samples was performed by a Leica TCS SP confocal microscope using the 63× apochromat lens.

### Immunoblotting

Proteins were resolved on 10% (w/v) SDS/PAGE [35], transferred to nitrocellulose membranes (porablot NCP, Macherey–Nagel) and immunoblotted as described previously [34]. The Ab reactivity was revealed either by a chromogenic reaction using 3,3′diaminobenzidine tetrahydrochloride (DAB) and H_2_O_2_ as substrates or by the ECL plus system (Amersham). In the latter case, nitrocellulose membranes were either analysed in a Phosphoimager or exposed to Kodak photographic films further developed with Kodak reagents. The ImageJ software [36] was used to process the film digital images.

### Preparation of *Leishmania* promastigote total membranes

Stationary phase wt and transgenic *L. donovani* (strain LG13) promastigotes (1.5–2×10^9^ cells), were harvested by centrifugation (1000 ***g***, 7 min, 4°C) and the cell pellets were suspended in PBS, pH 7.2. Subsequent procedures were performed at 4°C. The cell pellet was resuspended in lysis buffer (10 mM Tris/HCl, 125 mM sucrose, 3 mM MgCl_2_, 2 mM EDTA, pH 8) with proteolytic inhibitors, left on ice for 30 min and was then disrupted by a pre-chilled Dounce homogenizer. Cell disruption was followed with a microscope. After centrifugation (1000 ***g***, 10 min), the supernatant was further centrifuged at 200000 ***g*** for 1 h (SW41 Beckman rotor) and the pellet (membrane fraction) was resuspended in 50 mM Tris/HCl (pH 7.4) at a final volume 100–150 μl. Protein concentration was determined by the Bradford (Biorad) method.

### Detergent-based fractionation

Digitonin (Sigma) fractionation in *Leishmania* cells (∼2×10^9^ cells) was performed, as described previously, using progressively increased detergent concentrations (i.e 20 μM, 200 μM, 1 mM or 10 mM) [37]. Fraction 5, corresponding to the pellet left after treatment with 10 mM digitonin, was further solubilized with 1% (v/v) Triton X-100 (1 h, 4°C) and the soluble (F5 S) and insoluble (F5 P) fractions were recovered by centrifugation (20000 ***g***, 20 min, 4°C). Protein fractions (F1–F4) were obtained after acetone precipitation (overnight −20°C) and analysed by Western blot.

HeLa cells were solubilized in PBS with 1% (v/v) Triton X-100 plus protease inhibitors for 1 h at 4°C with rotation. The supernatant enriched in membrane proteins soluble in 1% (v/v) Triton X-100 was obtained by centrifugation (18000 ***g***, 25 min, 4°C) and further analysed by Western blot.

### Deglycosylation

Digitonin F5 S protein fraction (∼25 μg) from stationary phase *L. donovani* r*Ld*MAcP–mRFP1 promastigotes was digested (2 h, 37°C) with 500 units of endoglycosidase F (EndoF; New England Biolabs) according to the manufacturer's instructions.

A 1% (v/v) Triton X-100 soluble protein fraction (∼15 μg) of HeLa–r*Ld*MAcP–His cells was digested with 500 units of EndoF, as described above.

### Measurement of acid ecto-phosphatase activity

Acid ecto-phosphatase activity in live cells was routinely assayed at 37°C for 30 min using the phosphatase substrate *p*-nitrophenyl phosphate (*p*NPP; 10 mM) in a 90 mM citrate buffer at pH 4.8 (Sigma), with or without sodium tartrate [L(+)-tartaric acid; Merck; 2.5 mM or 5 mM for mammalian or parasite cells respectively]. Briefly, (i) *Leishmania* stationary phase promastigotes were washed twice with 100 mM Hepes and the cell pellet was resuspended in the substrate solution. The reaction was terminated by adding two volumes of 0.5 N NaOH. Subsequently, the parasites were removed by centrifugation (2000 ***g***, 5 min) and the absorbance of the reaction product [*p*-nitrophenolate (*p*NP)] was measured in the supernatant at 405 nm; (ii) HeLa cells expressing the r*Ld*MAcP–His chimera or the empty vector were incubated with substrate buffer and the reaction was terminated. The absorbance of *p*NP in the extracellular medium was measured as above.

The enzyme activity (*A*_405_ values) was normalized for the mean number of cells/ml used for each independent experiment (*Leishmania* or HeLa cells) and for the transfection efficiency, estimated by IF with the anti-histidine mAb (HeLa cells). Viability of the cells was assessed before and after incubation with the reaction medium, qualitatively by visual inspection of the parasite motility under the microscope and quantitatively by 0.4% (w/v) Trypan Blue staining.

### Macrophage infection assay

J774 mouse macrophages were seeded at ∼2.5×10^5^ cells/well on 10-mm coverslips and a suspension of transgenic *L. donovani* stationary phase promastigotes, with enhanced virulence (see below) was added at 20:1 ratio (parasites/macrophages) for spontaneous uptake (1 h, 37°C, 5% CO_2_). Unbound parasites were removed by extensive washing and the incubation continued for 3, 23 and 47 h and longer. Phagocytosis was stopped by fixation with paraformaldehyde [4% (w/v) in PBS]. Non-internalized parasites were labelled with mouse anti-LG13 pAb (1:250) and anti-mouse Alexa Fluor® 546. Non-bound Abs were removed by washing with PBS and the bound Abs were subjected to a second fixation step. Labelling of the internalized parasites was then performed by incubation with the same primary and different secondary (anti-mouse Alexa Fluor® 488) Ab solutions both in the presence of 0.1% (v/v) TritonX-100. Coverslips were processed for microscopy analysis as described above. Non-internalized parasites were visualized in the red and green fluorescence (FL) channels whereas internalized parasites were visualized in the green channel. Z-stacks from 1 μm thick optical sections were acquired. Parasites and macrophages were enumerated in the max projections by the use of the Icy digital image analysis software [38]. The virulence index was calculated as previously described [19] [virulence index=number of internalized parasites × number of infected macrophages/number of examined macrophages].

*L. donovani* promastigotes (wt and transgenic) were kept virulent by isolation from the spleen of infected BALB/c mice, susceptible to *Leishmania* infection, according to published protocols [14]. In the case of the transgenic parasites, Nourseorthicin (100 μg/ml) was added in the culture medium until the emergence of promastigotes. Animals were obtained from the breeding unit of the Hellenic Pasteur Institute and all experimental procedures were approved as above.

### *Leishmania* survival in macrophages

Macrophages (∼2.5×10^6^ cells) were incubated the day before the experiment for 1 h at 37°C with stationary phase *Leishmania* promastigotes (ratio 20:1, parasites/macrophages). Non-internalized parasites were then removed by excessive washing, fresh medium was added and the incubation was continued for 1–3 days. At the end of the incubation period, the macrophages were washed twice with PBS and lysed with 0.01% (v/v) SDS in PBS (30 min, RT), to release internalized parasites. Broken macrophages were removed by centrifugation (250 ***g***, 5 min). The parasites in the supernatant were collected by centrifugation (2000 ***g***, 10 min) and labelled [30 min, room temperature (RT)] with Cell Tracker Green (CMFDA, Molecular Probes®; 5 μM in PBS). Finally, the cells were washed with PBS and analysed by FACS (FACS Calibur, BD Biosciences). To define the population of apoptotic/necrotic cells incorporating CMFDA, the parasites were treated with 4 mM H_2_O_2_ for 12 h before labelling [39]. On the basis of this analysis the histogram gates for live or apoptotic/necrotic cell population were set as M3 and M2 respectively.

### Protein structure modelling

Structure models of the *Ld*MAcP (residues 26–313) and *Ld*SAcP1 (residues 26–392) were generated with the homology modelling program Swiss Model [40] based on comparison with the structure of the human prostatic acid phosphatase (hPAP; residues 1001–1343). PROCHECK [41] and Verify3D [42] software was used to validate the model structure. Structures were visualized and molecular images were generated using Pymol (DeLano Scientific http://www.pymol.org/). Disorder predictions on the basis of protein primary sequence were carried out with the network program PONDR-VXLT [43,44]. Phylogenetic trees were generated by the Phylip algorithm (http://evolution.genetics.washington.edu/phylip.html).

### Bioinformatics and statistical analysis

**Figure 1:**
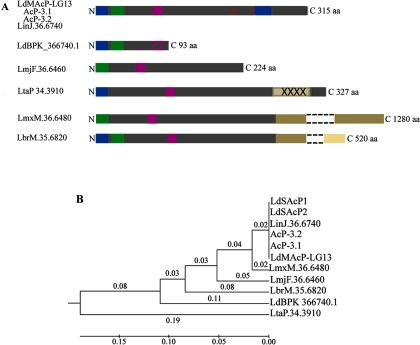
Protein sequence comparison of *Ld*MAcP orthologues in *Leishmania* sp (**A**) Schematic representation of the domain organization of *Ld*MAcP orthologues based on the ClustalW multiple sequence alignment (Supplementary Figure S1). High sequence homology is represented by the same colour in the bars. Red vertical lines indicate the sequence polymorphisms in *L. donovani* strains, dark blue boxes the putative SP (Met^1^-Ala^23^) and TM domain (Leu^274^-Tyr^302^) of *Ld*MAcP, the green box the HAcP signature motif and the purple box (Glu^73^-Leu^84^) the peptide sequence used to generate the anti-*Ld*MAcP mAb. The empty purple box signifies the absence of the Glu^73^-Leu^84^ sequence. X stands for non-identified aa. Dashed lines indicate repetitive sequence omitted due to size constraints of the graph. (**B**) Rooted phylogenetic tree (UPGMA) from a multiple sequence alignment of the *Ld*MAcP orthologues from *Leishmania* sp. The sequences of *Ld*SAcP1 (AAC79513) and *Ld*SAcP2 were also included in the analysis. The numbers indicate branch lengths.

The algorithms used were as follows: (1) TritrypDB BLAST (Basic Local Alignment Search Tool; http://tritrypdb.org/tritrypdb/showQuestion.do;jsessionid=16569DCDE981907D9-D1B21D0127473C4?questionFullName=UniversalQuestions.UnifiedBlast); (2) SignalP 4.1 Server (http://www.cbs.dtu.dk/services/SignalP/); (3) ClustalW2 (http://www.ebi.ac.uk/Tools/msa/clustalw2/); (4) BioEdit Sequence Alignment Editor, version 7.0.9.0 (Ibis Biosciences); (5) TMHMM Server v. 2.0 (http://www.cbs.dtu.dk/services/TMHMM-2.0/); (6) Icy digital image analysis (http://icy.bioimageanalysis.org/) 7) The phylogenetic tree (Figure 1B) was constructed using the MEGA (Molecular Evolutionary Genetics Analysis tool; http://www.megasoftware.net/).

For BLAST search for *Ld*MAcP orthologues in *Leishmania* sp. genomes and for primer design (Supplementary Table S1) we used the *Ld*MAcP sequence originating from the Sudanese *L. donovani* strain 1S-CL2D [20]. Graphs and statistical significance were prepared and analysed using the GraphPad Prism Software 5.01 (GraphPad). The paired Student's *t* test was used to evaluate statistical significance among groups.

## RESULTS AND DISCUSSION

### The *Ld*MAcP ecto-phosphatase is specific for the *L. donovani* complex and presents strain polymorphisms

The original identification of the *Ld*MAcP protein [20] was followed by a study indicating that its sequence is conserved among all pathogenic *Leishmania* sp. [45]. To confirm this, a search for *Ld*MAcP homologues was conducted in the available genome sequences of several *Leishmania* sp. The predicted *Ld*MAcP protein sequence from *L. donovani* (strain LG13) generated in the present study, was almost identical with the AcP-3.1 (AF149839.1) and AcP-3.2 *Ld*MAcP sequences and with the *L. infantum* orthologue (LinJ.36.6740; Figure 1; Supplementary Figure S1). Surprisingly, the *Ld*MAcP orthologue from the Nepalese *L. donovani* LDBPK282A1 strain (LdBPK_366740.1) [46] turned out to be a truncated form (93 aa) containing only the 72 N-terminal aa from the Ethiopian and Sudanese orthologues and no C-terminal transmembrane domain (TM; Figure 1A, Supplementary Figure S1). Identification of the LdBPK_366740.1 gene locus on a circular episome, implicated in the gene dosage effect associated with drug resistance, suggests that expression of this gene might be regulated by the parasite by several environment dependent mechanisms [46]. This could also explain the considerable variability in this enzyme activity previously detected [45] in different *Leishmania* sp. and geographic isolates of the same species.

Comparison of the *L. donovani* MAcP with the *L. major* Friedlin (LmjF.36.6460) and the *L. tarentolae* Parrot-Tarll (LtaP34.3910) orthologues showed 76.3% and 65.4% identity (ClustalW2 analysis) respectively, in the predicted protein sequence and significant differences in the predicted protein domain organization (Figure 1; Supplementary Figure S1). The *L. major Ld*MAcP orthologue lacks the predicted N-terminal endoplasmic reticulum (ER) signal peptide (SP) sequence and is not predicted to contain a TM domain at its C-terminus (TMHMM bioinformatics tool; result not shown). It is therefore predicted to be an intracellular soluble enzyme. Examination of the LmjF.36.6460 locus sequence 400 bp upstream the 5′-end of the predicted open reading frame (ORF), revealed a sequence almost identical with the 84 bp upstream of the second ATG codon of the *Ld*MAcP*–*LG13 sequence. However, a 2 bp deletion 63 bp downstream of the first ATG codon, changes this ORF and introduces stop codons. The new ORF begins at the second Met^29^. To confirm this, we amplified by PCR the LmjF.36.6460 sequence from *L. major* Friedlin genomic DNA using primer sequences (Supplementary Table S1) located 100 bp upstream and downstream of the 5′- and 3′-non translated regions of this ORF. Sequencing of the generated PCR product (result not shown) confirmed the ORF sequence for LmjF.36.6460 published in the TriTrypDB. Additionally, it confirmed a 65 aa deletion with respect to the *Ld*MAcP–LG13 sequence (residues 158–223), a region in the predicted extracellular globular domain. This may result in changes in the folding of the protein and a possible loss of catalytic activity.

On the other hand, the LtaP34.3910 ORF codes for a protein more divergent (Figure 1B) and 10 residues longer than the *Ld*MAcP. It contains 150 non-identified nucleotides at the 3′-end and thus it is not clear whether the predicted sequence harbours a TM domain (Supplementary Figure S1, Figure 1A, X symbols) or a stop codon in the non-sequenced region, upstream of the predicted stop codon. To clarify this, we generated and sequenced a PCR product of the LtaP34.3910 ORF extending by 97 bp downstream of the predicted stop codon (Supplementary Table S1). After several efforts of sequencing, we could not clarify further the structural features of this region (result not shown).

The *Leishmania braziliensis* (LbrM.35.6820) and *Leishmania mexicana* (LmxM.36.6480) sequences retrieved as *Ld*MAcP orthologues in the BLAST search are by 205 aa and 965 aa longer respectively (Figures 1A and 1B). The LbrM.35.6820 sequence lacks the C-terminal predicted TM domain representing a secreted form and an orthologue of the *Ld*SAcPs [21]. The LmxM.36.6480 sequence additionally lacks the SP and it may represent an intracellular form of the protein.

The above *in silico* analysis showed that the *L. major, L. braziliensis, L. mexicana* and *L. tarentolae* MAcP orthologues, are not predicted to localize at the parasite surface membrane and could represent intracellular or secreted forms of the protein. It is interesting to note that *L. tarentolae* infects reptiles and is considered to be non-pathogenic to mammals in contrast to *L. donovani* and the other anthroponotic *Leishmania* sp. The differences in the biology of the respective species which reflect adaptations to different host organisms are depicted in a genome sequence analysis of the *L. tarentolae* Parrot-Tarll and in a comparison with the already sequenced anthroponotic species [47]. The absence in *L. tarentolae* of several genes coding for surface proteins or proteins involved in endocytosis or exocytosis may have important consequences for the ability of *L. tarentolae* to survive as an intracellular parasite within macrophages and evade the immune system in mammalian hosts.

In conclusion, *Ld*MAcP, as a membrane bound enzyme with predicted extracellular orientation seems to be present only in the *L. donovani* complex in which it presents few strain polymorphisms. This finding challenges an earlier report suggesting that the *Ld*MAcP is conserved in *Leishmania* sp. [45]. However, in the latter study, the probe used for the Southern blot analysis corresponded to a short region of the *Ld*MAcP gene sequence that is indeed conserved among the *Leishmania* sp.

### *In silico* 3D modelling of the *Ld*MAcP structure; comparison with the secreted acid phosphatase *Ld*SAcP1

The *Ld*MAcP was initially identified [20] as a protein highly homologous to the *Ld*SAcPs [21], encoded by two tandemly arrayed genes located in the *L. donovani* chromosome 36. The *Ld*MAcP and *Ld*SAcPs share a surprisingly high sequence identity in their first 274 N-terminal aa [20]. This could represent either a functional redundancy or different functional roles for these three enzymes. We proceeded to analyse their predicted 3D structures in order to provide insight into this question.

**Figure 2:**
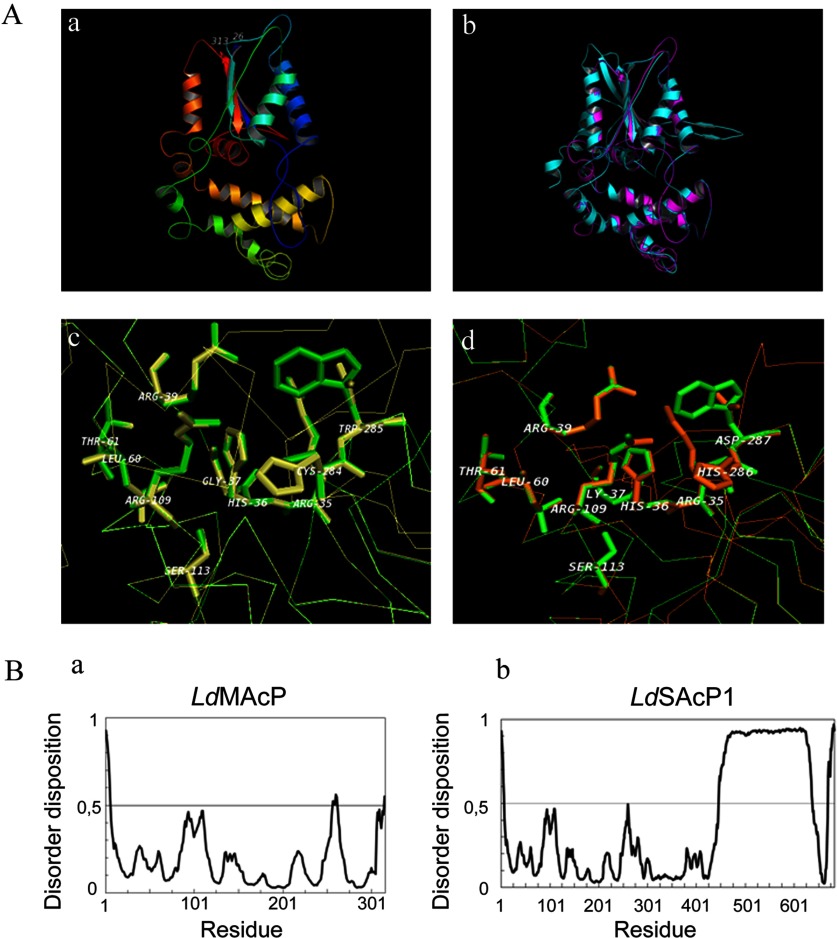
*In silico* structural analysis of the *Ld*MAcP and *Ld*SAcP1 (**A**) 3D-molecular modelling of the *Ld*MAcP (and *Ld*SAcP1 proteins. (**a**) *Ld*MAcP 3D-molecular model. Each secondary structure element is presented in a different colour. (**b**) Superimposition of the *Ld*MAcP and *Ld*SAcP1 molecular models (*Ld*MAcP in purple, *Ld*SAcP1 in cyan). (**c**) molecular model of the *Ld*MAcP catalytic site (green) superimposed on the structure of the hPAP catalytic site (yellow). (**d**) Superimposition of the molecular models for the *Ld*MAcP (green) and *Ld*SAcP1 (orange) catalytic sites. Critical residues are indicated. (**B**) PONDR diagram of *Ld*MAcP (**a**) and *Ld*SAcP1 (**b**). Residues with PONDR score >0.5 are expected to be in a disordered structure, residues with score <0.5 are predicted to be in an ordered structure. Values close to 1 are strong indicators of disorder.

The high sequence identity with the human hPAP [∼24% for the region that has a resolved crystal structure (residues 33–374)], allowed us to construct reliable 3D structure models for the *Ld*MAcP (residues 26–313) and the *Ld*SAcP1 (residues 26–392) (Figures 2a and b). These models were generated by comparative modelling based on the structure of hPAP and were validated by established structure validity criteria. The RMSDs over the Cα atoms between the model and the template were 0.32 Å for *Ld*SAcP1 and 0.35 Å for *Ld*MAcP. For the backbone atoms RMSDs were 0.47 and 0.51 Å respectively. More than 90% of the residues were found in the allowed Ramachandran plot regions.

As already mentioned, all three proteins belong to the family of HAcPs [22]. The second HAcP signature sequence (Ala^285^ to Thr^296^) present in the *Ld*SAcPs is absent from the 948 bp ORF-deduced *Ld*MAcP protein. A dendrogram representation of selected structures from HAcP family members (Supplementary Figure S2) suggests that the *Ld*MAcP and the *Ld*SAcPs belong to branch 2 HAcPs [22] and are more homologous and evolutionarily related to the human and rat PAPs. Branch 2 HAcPs appear to enter the secretory pathway. Some enzymes remain in the ER, others are found at the cell surface, periplasm or cell wall and others are simply secreted [22].

Residues conserved throughout the HAcP superfamily are also conserved in *Ld*MAcP and *Ld*SAcPs, with the exception of His^286^ and Asp^287^, which are missing in *Ld*MAcP (Figures 2c and d). These two residues are considered important for the formation of the catalytic site and are absolutely conserved in the family. His^286^ is supposed to close the catalytic site and offers additional electrostatic support due to its positive charge, whereas Asp^287^ is thought to act as the proton donor in the catalytic cycle. Histidine deletion abolishes catalytic activity, but aspartic acid might only facilitate contact with the substrate [48]. The different structural features of *Ld*MAcP in this specific region result in a more ‘open’ and less-charged active site that could accommodate a much bulkier substrate, such as phosphorylated proteins, and facilitate interactions with more hydrophobic parts of the substrate. In *Ld*MAcP the two corresponding residues as shown from the structural alignment are cysteine and tryptophan, residues which are much more hydrophobic than histidine and aspartate. However, these residues are predicted to be in the TM domain of *Ld*MAcP. It seems that this protein has to be bound to the membrane at the expense of losing catalytic residues.

To gain further insight into the structural differences of the two molecules that could explain their difference in localization (and possibly their functionality) we analysed their sequences using the well-established neural network program PONDR [43,44], which gave a convincing prediction of intrinsic disorder for the 300 aa C-terminal part of *Ld*SAcP1 missing in the *Ld*MAcP sequence (Figures 2e and 2f). The common part of the two molecules, on the other hand, is predicted to be globular and well-folded, as expected. The *Ld*SAcPs, with a predicted highly disordered C-terminal half, may possess high specificity, low affinity and an unusual diversity of interactions with a multitude of partners inside or outside the host cells, whereas the membrane bound *Ld*MAcP could recognize a more limited range of substrates. It is known that several viruses hijack their host cells by using intrinsic disorder and the functional advantage of conformational adaptability that it confers to mimic key regulatory proteins that are themselves intrinsically disordered [49]. Identification of natural host substrates for *Ld*MAcP and *Ld*SAcPs will provide answers as to whether these proteins play a role in the subversion of the host macrophage signalling mechanisms by *L. donovani* parasites. Interestingly, HAcP from the category A pathogen *Francisella tularensis* (FtHAP), implicated in the intra-macrophage survival and virulence of the bacterium, has the same fold as the hPAP and belongs to the branch 2 family HAcPs [50] similarly to the *L. donovani Ld*MAcP and *Ld*SAcPs.

Thus, the *in silico* structural analysis of the *Ld*MAcP and *Ld*SAcPs sequences suggests differences in the functionality of the two enzymes.

### The *Ld*MAcP and r*Ld*MAcP–mRFP1 proteins have ER and surface membrane localization in stationary phase *L. donovani* promastigotes

**Figure 3:**
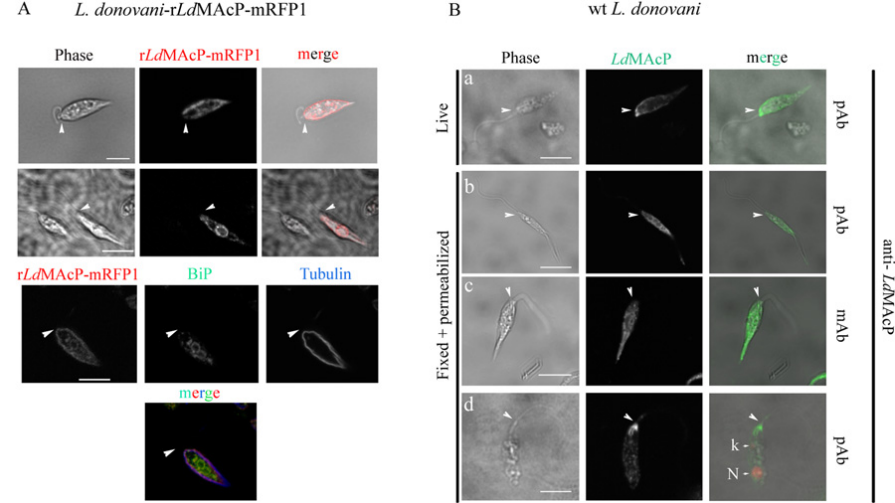
Localization of *Ld*MAcP in transgenic *L. donovani* r*Ld*MAcP–mRFP1 and wt *L. donovani* stationary phase promastigotes (**A**) r*Ld*MAcP–mRFP1 FL visualized by confocal microscopy. IF co-staining of fixed cells permeabilized with 0.1% (v/v) Triton X-100 was performed with anti-BiP (1:500) pAb and anti-α-tubulin (1:300) mAb followed by secondary Abs conjugated to Alexa Fluor® 488 and Alexa Fluor® 633 respectively. Acquisition of green and far red FL images performed simultaneously was followed by acquisition of the red (mRFP1) FL. (**B**) The endogenous *Ld*MAcP was visualized by IF with the anti-*Ld*MAcP mAb and pAb in live (stained at 4°C) and fixed permeabilized wt *L. donovani* promastigotes either stably cultured at 25°C (**a**–**c**) or incubated for 1 h at 37°C prior to fixation (**d**). Nuclear (N) and kinetoplast (k) DNA was stained with PI (**d**). Images were acquired by z-scanning performed at 0.5 μm step size. Single optical sections are shown in all panels. Phase contrast and FL images are presented in black and white (BW). Merged images of FL and phase contrast at 50% transparency are presented in colour. Arrow heads point to the beginning of the flagellum. Scale bar 4 μm.

In order to confirm the predicted localization of the *Ld*MAcP on the parasite surface membrane [20] where it could confer the acid ecto-phosphatase activity detected in the *L. donovani* cells, we expressed episomally in *L. donovani* promastigote parasites the full length protein as a recombinant C-terminally tagged *Ld*MAcP–mRFP1 chimera. The localization of r*Ld*MAcP–mRFP1 was followed by direct visualization of the mRFP1 FL in the *L. donovani* r*Ld*MAcP–mRFP1 transgenic promastigotes by confocal microscopy. The r*Ld*MAcP–mRFP1 had a surface and perinuclear ER-like localization (Figure 3A) further confirmed by co-staining with the anti-α-tubulin mAb, staining the subpellicular microtubules, and the anti-BiP pAb specific for the BiP/GRP78 Leishamnial ER chaperone [51] respectively (Figure 3A, bottom panels). No FL signal was observed in the flagellum. Similar localization pattern, as well as staining at the flagellar pocket (FP) [52], was revealed when wt *L. donovani* (strain LG13) promastigotes permeabilized with Triton X-100 were stained by indirect IF with the anti-*Ld*MAcP mouse mAb or pAb (Figure 3B). The FP staining was even stronger when parasites were incubated at 37°C for 1 h prior to fixation and staining (Figure 3B, bottom panels) indicating higher levels of *Ld*MAcP secretion to the parasite plasma membrane at the body temperature of the mammalian host. It is important to note that FP is the domain of the *Leishmania* cell that supports exocytosis and endocytosis. The FP membrane is an obligatory intermediary ‘station’ for the trafficking of membrane bound molecules between intracellular membranes and the cell surface and vice versa. From this ‘station’, membrane bound molecules move by lateral diffusion to the surface of the cell body and the flagellum [16,53].

To confirm the topology of the putative globular/catalytic domain of *Ld*MAcP we performed immunostaining of live promastigotes at 4°C using anti-*Ld*MAcP pAb. As shown in Figure 3(B) (top panels), the antibodies recognized epitopes on the entire surface of the parasites and in the FP, indicating access of the extracellular Ab to this site. This staining pattern was consistent with the predicted extracellular localization of the *Ld*MAcP epitopes recognized by the anti-*Ld*MAcP Abs (Figure 1A; Supplementary Figures S1 and S3). Staining with mouse pre-immune serum confirmed the specificity of the anti-*Ld*MAcP mouse sera (result not shown).

Cumulatively, we have concluded that the full-length *Ld*MAcP protein shows typical localization for type I membrane proteins following the secretory pathway of *Leishmania* parasites. Interestingly, in higher eukaryotes, the lysosomal acid phosphatase that belongs to branch 2 HAcPs, as does *Ld*MAcP, is unusually transported as a type I membrane protein from the ER, via the plasma membrane, to the lysosome [54] where its luminal portion is slowly proteolytically cleaved and it is released from the membrane.

Trafficking of surface membrane bound enzymes via endocytosis to the lysosome or lysosome-like compartments takes place in protozoan parasites of the *Trypanosomatidae* family [16,53], as well. One of the ways by which resident lysosomal proteins and lipids are delivered to the lysosome is by initial delivery to the FP and subsequent internalization to the lysosome via the tubular endosome complex. Additionally, endocytosis of surface membrane proteins [55] that takes place by a highly complex and developmentally regulated endocytic network [56], is vital for nutrient uptake [55] and evasion of the immune response [57]. Interestingly, in *L. mexicana*, a type I membrane bound acid phosphatase [58] that is not an *Ld*MAcP orthologue, traffics between the cell surface, the endosomes and the multivesicular lysosome when it is overexpressed. The same was observed in the bloodstream form of *Trypanosoma brucei*, for a membrane-bound acid phosphatase involved in endocytosis/exocytosis and in parasite differentiation to the insect stage [59]. Consistent with these results, in another protozoan human pathogen, *Plasmodium falciparum*, identification of surface proteins by proteomic analysis in the food vacuole, a lysosome-like organelle [60], suggests the existence of a trafficking route between the plasma membrane and the food vacuole.

Therefore, we cannot exclude the possibility that *Ld*MAcP follows a similar trafficking path from the FP to the lysosomes or to the acidocalcisomes (lysosome like compartments) of *Leishmania* [61–63].

### The endogenous *Ld*MAcP and the recombinant r*Ld*MAcP–mRFP1 proteins are detected in *L. donovani* plasma membrane enriched fractions and are N-linked glycosylated

To detect biochemically the expression of recombinant and endogenous *Ld*MAcP polypeptides in *Leishmania* cells we prepared total membrane fractions from transgenic *L. donovani* r*Ld*MAcP–mRFP1 and wt *L. donovani* (strain LG13) stationary phase promastigotes. The r*Ld*MAcP–mRFP1 and endogenous *Ld*MAcP were detected by both the anti-mRFP and the anti-*Ld*MAcP Abs by Western blot analysis.

**Figure 4:**
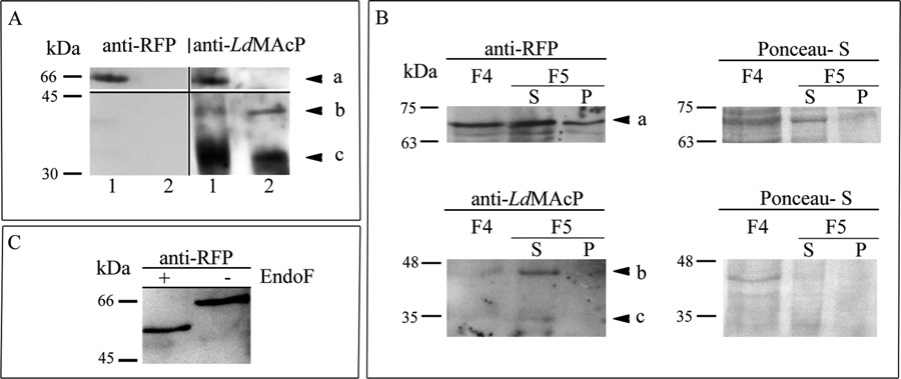
Biochemical detection of endogenous *Ld*MAcP and r*Ld*MAcP–mRFP1 chimera in *L. donovani* stationary phase promastigotes (**A**) *L. dovovani* wt (lane 2) or *L. donovani* r*Ld*MAcP–mRFP1 (lane 1) total membrane fractions (∼15 μg/lane) were immunoblotted with purified rabbit anti-mRFP pAb (0.4 μg/ml) and after stripping with mouse anti-*Ld*MAcP mAb. Black arrow heads on the right indicate the protein bands corresponding to r*Ld*MAcP–mRFP1 (**a**) and *Ld*MAcP (**b** and **c**). (**B**) Equal volumes of *L. donovani* r*Ld*MAcP–mRFP1 digitonin protein fractions F4, F5S and F5P (Experimental) were immunoblotted with anti-mRFP pAb and anti-*Ld*MAcP mAb, as above. Ponceau-S of respective membrane regions (right) are shown as loading indicators. Black arrow heads indicate the protein bands corresponding to r*Ld*MAcP–mRFP1 (**a**) and *Ld*MAcP (**b** and **c**). (**C**) Deglycosylated r*Ld*MAcP–mRFP1. Digitonin protein fraction F5 S (25 μg/lane) treated (+) or not treated (−) with 500 units of EndoF was immunoblotted with anti-mRFP pAb. Results are from one representative experiment. Molecular masses are indicated in kDa.

In membranes prepared from the *L. donovani* r*Ld*MAcP–mRFP1 promastigotes, a protein of apparent *M*_r_=66000 was detected (Figure 4A, lane 1, arrow head a). This band corresponds to r*Ld*MAcP–mRFP1 [predicted *M*_r_=61706 with the SP or *M*_r_=59470 after cleavage of the SP) since it was revealed by both the anti-RFP and the anti-*Ld*MAcP Abs and was absent from the wt parasite membranes (Figure 4A, lane 2). The anti-*Ld*MAcP mAb detected in both the *L. donovani* r*Ld*MAcP–mRFP1 and the wt *L. donovani* membranes, a prominent *M*_r_ ∼ 35000 broad band corresponding to the calculated *M*_r_=35192 of the native *Ld*MAcP (Figure 4A, lanes 1 and 2, arrow c). This band could also contain the *Ld*MAcP after cleavage of the SP sequence (1–23 aa) smaller by ∼2.2 kDa. Interestingly, a band of *M*_r_=40000 (Figure 4A, lanes 1and 2, arrow b) was also detected. A similar protein pattern was identified by Western blot analysis of subcellular protein fractions produced by subjecting *L. donovani rLd*MAcP–mRFP1 stationary phase promastigotes to digitonin fractionation (Experimental) and further solubilization of ‘Fraction 5’ with 1% (v/v) Triton X-100 (Figure 4B, F5 S). Finally, the recombinant r*Ld*MAcP–His protein expressed in bacteria, migrated in SDS/PAGE with an apparent *M*_r_ ∼ 37000, corresponding to its predicted *M*_r_=36775 and was detected with all anti-*Ld*MAcP Abs prepared in the present work (Supplementary Figure S3). These results suggest that the r*Ld*MAcP–mRFP1 and the endogenous *Ld*MAcP proteins produced in *L. donovani* promastigotes are subjected to post-translational modifications.

N-glycosylation could account for the difference between the apparent and predicted molecular mass of the recombinant and endogenous *Ld*MAcP forms detected in the present study (Figures 4A and 4B). This hypothesis was strengthened by the fact that the *Ld*MAcP aa sequence contains five putative N-glycosylation sites (Asn^44^, Asn^96^, Asn^135^, Asn^219^ and Asn^245^) [20]. We therefore explored this possibility by treating the digitonin fraction F5 S with EndoF (Figure 4C). The r*Ld*MAcP–mRFP1 protein was indeed found to be N-glycosylated, as indicated by its reduced mobility in SDS/PAGE by ∼5–7 kDa upon EndoF treatment. This corresponds to the difference between the apparent and calculated molecular masses observed for r*Ld*MAcP–mRFP1 and *Ld*MAcP (Figures 4A and 4B).

Efficient glycosylation could regulate the transport of *Ld*MAcP to the plasma membrane and/or the stability of the enzyme. It is also possible that *Ld*MAcP transport to the cell surface could be regulated by additional post-translational modifications, e.g. phosphorylation. Residue Tyr^302^ at the C-terminal cytoplasmic tail of the protein is conserved in both *Ld*MAcP and *Ld*SAcPs. *In silico* analysis of the *Ld*MAcP sequence showed that it contains putative phosphorylation and myristoylation motifs (result not shown), which should be confirmed experimentally.

### Overexpression of *Ld*MAcP in *L. donovani* promastigotes results in an increased tartrate resistant acid ecto-phosphatase activity

To assess whether the surface expression of r*Ld*MAcP–mRFP1contributes to acid ecto-phosphatase activity of the *L. donovani*-r*Ld*MAcP–mRFP1 parasites, we measured the dephosphorylation of the generic substrate *p*NPP at pH 4.8 in live parasites at the stationary phase of growth at 37°C, the temperature of the mammalian host in which the parasites are inoculated by the sandfly vector during the blood meals. Firstly, we compared this enzymatic activity to that of the parental wt *L. donovani* population and found it by ∼2.5-fold higher (Table 1). This result was in accordance with the qPCR analysis (Experimental) results which showed that the *Ld*MAcP gene was overexpressed by ∼4-fold in *L. donovani* r*Ld*MAcP–mRFP1 parasites with respect to the wt parental strain. Additionally, cumulative results from five independent experiments showed that in *L. donovani* r*Ld*MAcP–mRFP1 promastigotes the tartrate resistant acid ecto-phosphatase activity was more than 10-fold higher than in the transgenic *L. donovani*-Lexsy-sat (Table 1) promastigotes. Analogous results were obtained when the total acid phosphatase activity was measured in the digitonin fraction F5 S obtained from both *L. donovani* Lexsy-sat and *L. donovani* r*Ld*MAcP–mRFP1 parasites (result not shown).

**Table 1:** Acid ecto-phosphatase activity in live, stationary phase transgenic *L. donovani* r*Ld*MAcP–mRFP1 and *L. donovani* Lexsy-sat promastigotes Enzymatic activities assayed in the absence or presence of 5 mM sodium tartrate are expressed as absorbance (405 nm) of *p*NP/10^7^ cells/ml. Results are mean values ± S.D.s from five independent experiments. *P<0.05 compared with corresponding control values (*L. donovani* Lexsy-sat), using a two-tailed paired Student's *t* test.

	Ecto-phosphatase activity (A_405_/10^7^ cells × ml^−1^)	
Trangenic population		+5 mM sodium tartrate
*L. donovani* Lexsy-sat	42±15	22±11
*L. donovani* r*Ld*MAcP–mRFP1	455±216*	275±192*
*L. donovani* (strain LG13)	142±57*	63±26*

It is worth mentioning that the levels of acid ecto-phosphatase activity measured in our study varied considerably depending on the parasites’ phase of growth but, also among different experiments when parasites used were at the same phase of growth. This variation is depicted by the S.D.s in Table 1. Finally, we confirmed that the enzymatic activity measured in our assays with live cells was due to acid ecto-phosphatase(s) and not to released enzymes by incubating the same number of parasites used in the assay with the reaction medium at 37°C for 30 min, removing the parasites by centrifugation and testing for *p*NPP hydrolysis in this medium devoid of cells [64]. The acid phosphatase activity detected thereby was at background levels (result not shown).

Interestingly, overexpression of *Ld*MAcP in the *L. donovani Ld*MAcP–mRFP1 parasites resulted in a ∼25% growth advantage at late stationary phase as compared with the control *L. donovani* Lexsy-sat population (result not shown). It is possible that when nutrients are limited at high parasite density, the higher levels of *Ld*MAcP activity provide parasites with an advantage in growth as previously suggested for the role of ecto-phosphatases in the proliferation of *Leishmania amazonensis* [9,65].

Thus, expression of the r*Ld*MAcP–mRFP1 at the parasite surface confers tartrate-resistant acid phosphatase activity, a result suggesting that the endogenous *Ld*MAcP is a tartrate resistant ecto-phosphatase. It would be extremely interesting, although technically more difficult, to investigate the *Ld*MAcP activity in *L. donovani* amastigotes that reside and multiply within the acidic host cell phagolysosome. Thus far, ecto-phosphatase activity in amastigotes that would suggest involvement of this enzymatic activity in the parasite's intracellular survival has been demonstrated directly in tissue derived *L. mexicana* amastigotes [66] and indirectly in lesion-derived and axenic *L. amazonensis* amastigotes [67]. Treatment of live *L. amazonensis* amastigotes with sodium orthovanadate, an inhibitor of acid and phosphotyrosine protein phosphatases, resulted in impairment or reversion of the amastigote-induced tyrosine dephosphorylation in amastigote-infected macrophages, suggesting that an ecto-phosphatase activity exists on the amastigotes’ surface.

### Tartrate resistant acid ecto-phosphatase activity varies significantly among different *Leishmania* sp.

As mentioned above, sequence comparison of the *Ld*MAcP protein with its orthologues from different *Leishmania* sp., revealed important species-dependent sequence and domain organization differences (Figure 1; Supplementary Figure S1). We therefore investigated whether these differences are reflected in the acid ecto-phosphatase activity in intact *L. major* Friedlin, *L. infantum* (strain GH12) and *L. tarentolae* (Parrot strain) promastigotes, as compared with that of the *L. donovani* (strain LG13) promastigotes. The present study was performed with promastigote parasite cultures in the stationary phase of growth as above.

According to our results, the *L. infantum* showed similar levels of acid ecto-phosphatase activity to that of *L. donovani* and similar sensitivity to sodium tartrate inhibition (Tables 1 and 2). Surprisingly, the *L. major* Friedlin strain had dramatically higher activity, ∼7–8 fold, than the *L. infantum* and *L. donovani* strains respectively and this activity was >90% sodium tartrate resistant. Given that the *L. major Ld*MAcP orthologue is not predicted to be a surface membrane enzyme since it lacks the ER SP sequence and has no predicted TM (Figure 1; Supplementary Figure S1), we could infer that *L. major* expresses another enzyme conferring on its plasma membrane a very efficient tartrate resistant acid ecto-phosphatase activity. Furthermore, the non-pathogenic to humans *L. tarentolae* showed significantly lower (only ∼0.3-fold) acid ecto-phosphatase activity than the visceral *L. donovani* and *L. infantum* strains (Tables 1 and 2). It would be interesting to find out if the low ecto-phosphatase activity observed in the present study in *L. tarentolae* is due to the *Ld*MAcP orthologue LtaP34.3910 or to another ecto-phosphatase. Moreover, our results suggest that besides the *Ld*MAcP activity which appears to be resistant to inhibition by L-tartrate (Table 1), a tartrate sensitive ecto-phosphatase activity should be present as well on the *L. donovani* and *L. infantum* plasma membrane.

**Table 2:** Acid ecto-phosphatase activity in live stationary phase wt *Leishmania* sp. promastigotes Enzymatic activities were assayed and expressed as in Table 1. Results are mean values ± S.D.s from three independent experiments. *P<0.05 and **P<0.01 compared with corresponding control values (*L. donovani*, strain LG13), using a two-tailed paired Student's *t* test.

	Ecto-phosphatase activity (A_405_/10^7^ cells × ml^−1^)	
Wt strains		+5 mM sodium tartrate
*L. donovani* (strain LG13)	142±57	63±26
*L. infantum* (strain GH12)	168±49	88±16
*L. major* Friedlin	1113±188**	925±87**
*L. tarentolae* (strain Paroll)	46±26*	27±14*

All the above presented results on the *Leishmania* acid ecto-phosphatase activity are in accordance with previous studies reporting species specific and strain specific variability of acid phosphatase(s) in *Leishmania* sp. [18,19,45,63,68].

### The r*Ld*MAcP–His transiently expressed in mammalian cells is targeted to the plasma membrane and has an extracellular topology with acid ecto-phosphatase activity

To further study the *Ld*MAcP ecto-phosphatase activity, we transiently expressed in HeLa cells the r*Ld*MAcP–His chimera and investigated its localization and contribution to the acid ecto-phosphatase activity of transfected live cells.

**Figure 5:**
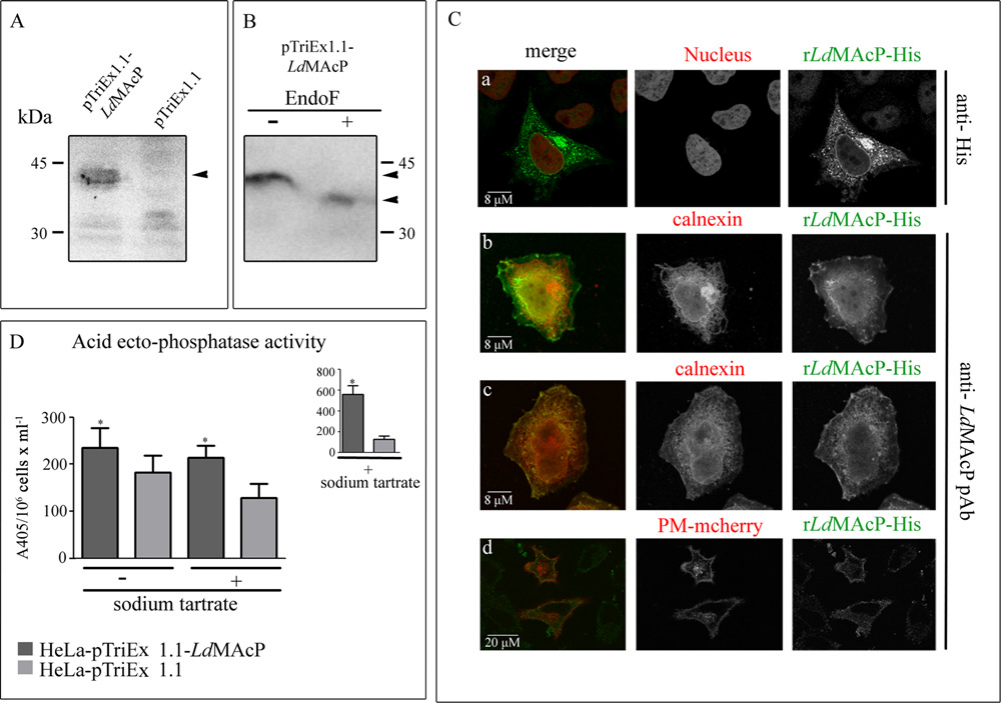
Heterologous expression of r*Ld*MAcP–His in HeLa cells HeLa cells were transfected with the pTriEx1.1–r*Ld*MAcP or the pTriEx1.1 plasmid and analysed 24 h post transfection by WB (**A** and **B**), IF (**C**) and for acid ecto-phosphatase activity (**D**). 1% (v/v) Triton X-100 soluble HeLa cell extract (∼10–15 μg/lane) was (**A**) immunoblotted with the mouse anti-His mAb (1 μg/ml), and (**B**) deglycosylated with (+) EndoF and immunoblotted with mouse anti-*Ld*MAcP pAb; (−) control sample without EndoF. Results are from one representative experiment. (**C**) r*Ld*MAcP–His was detected with anti-His mAb (**a**) and ER (**b** and **c**) with anti-calnexin rabbit pAb (4 μg/ml). Detection of plasma membrane was performed by co-transfection with the pDisplay-mcherry plasmid and staining of live cells with the anti-*Ld*MAcP mouse pAb (**d**). Nuclear DNA was stained with PI. Green (Alexa488) and red (Alexa546 or PI) FL are presented in black and white (BW) images and merged in colour. Images were acquired by z-scanning performed at 1 μm step size. Single optical sections are shown in all panels. (**D**) Enzyme activities (+ or − sodium tartrate) are expressed as absorbance (405 nm) of *p*NP/10^6^ cells/ml. The inset bar graph represents the enzymatic activity in the presence of sodium tartrate after normalization for transfection efficiency. Error bars are S.D.s from four independent experiments. *P<0.05, two-tail paired Student's *t* test.

The heterologous expression of the r*Ld*MAcP–His in HeLa cells, was initially confirmed by Western blot analysis with the anti-His mAb (Figure 5A) and the anti-*Ld*MAcP pAb (result not shown). As indicated in Figure 5(A), a protein band of *M*_r_ ∼ 42000 was detected in cells transfected with the pTriEx1.1–*Ld*MAcP plasmid. Interestingly, the mobility of this protein band was reduced to *M*_r_ ∼ 35000 upon EndoF treatment. This is approximately the calculated molecular mass of r*Ld*MAcP–His after removal of the ER SP. Therefore, as already demonstrated (Figure 4C) for the r*Ld*MAcP–mRFP1 expressed in *L. donovani*, the r*Ld*MAcP–His, expressed in HeLa cells this time, was also N-glycosylated (Figure 5B).

In the same cells, IF experiments using the anti-His mAb and anti-*Ld*MAcP pAb revealed that the r*Ld*MAcP–His was localized in the ER and plasma membrane (Figure 5C). The ER localization was confirmed by co-staining with an anti-calnexin Ab whereas the localization of r*Ld*MAcP–His to the plasma membrane was shown in cells co-transfected with the pTriEx1.1.–*Ld*MAcP and pDisplay-mcherry plasmids. The latter codes for a mCherry form targeted to the cell surface. The extracellular surface topology of the r*Ld*MAcP–His globular domain was confirmed in these cells by staining them live with the anti-*Ld*MAcP pAb (Figure 5C, bottom panel).

Finally, the extracellular localization of the r*Ld*MAcP*–*His active site was further confirmed by assaying the hydrolysis of the *p*NPP substrate in live transfected HeLa cells in the presence or absence of 2.5 mM sodium tartrate. HeLa cells transfected with the pTriEx1.1.–*Ld*MAcP plasmid exhibited significantly higher tartrate resistant acid ecto-phosphatase activity (Figure 5D). This difference was more pronounced (∼4–5-fold) when the data were normalized for the percentage of cells expressing r*Ld*MAcP–His (Figure 5D, inset). Acid phosphatase activity due to cell lysis, evaluated as described above for the *Leishmania* promastigotes, was found at background levels.

In summary, the above results, indicated that r*Ld*MAcP–His was successfully targeted by the HeLa secretory system to the plasma membrane with an active catalytic domain facing the extracellular side and confirmed, this time in a heterologous higher eukaryotic system, the enzymatic nature of *Ld*MAcP as an acid ecto-phosphatase.

### The *L. donovani* r*Ld*MAcP–mRFP1 promastigotes survive in J774 murine macrophages more efficiently than the *L. donovani* pLexsy-sat promastigotes

Acid ecto-phosphatase activity of *Leishamnia* sp. has been suggested to be implicated in the subversion of the host macrophage defence mechanisms by altering the levels of phosphorylated signalling molecules at the surface or inside the host cells [69]. Earlier studies have associated the extracellular surface acid phosphatase activity in *L. donovani* with the degree of promastigote virulence/infectivity [18,19]. However, in these studies this activity was measured either in whole-cell lysates or in crude membrane fractions and not in live parasites. Our data raise the intriguing possibility that the presence of the acid ecto-phosphatase activity in intact *L. donovani* promastigotes may be part of the pathogen's mechanism to manipulate a signal recognition system of the host macrophages in order to gain access and survive in its intracellular niche.

To investigate this hypothesis we used an *in vitro* macrophage cell culture infection system to examine whether the *L. donovani* r*Ld*MAcP–mRFP1 strain that showed 3- and 10-fold higher acid ecto-phosphatase activity (tartrate resistant) than the parental and the mock transfected *L. donovani* populations respectively (Tables 1 and 2), presented an infectivity advantage. We therefore compared the efficiency of *L. donovani* promastigotes carrying either the pLexsy-sat (*L. donovani* Lexsy-sat) empty vector or the pLexsy-sat–r*Ld*MAcP–mRFP1 (*L. donovani* r*Ld*MAcP–mRFP1) plasmid to infect J774 mouse macrophages in culture. The parasites used in these experiments had enhanced virulence (Experimental) and were used at the stationary phase of growth, as this population is enriched in metacyclics, the infectious parasitic form injected by the sandfly into the mammalian host.

**Figure 6:**
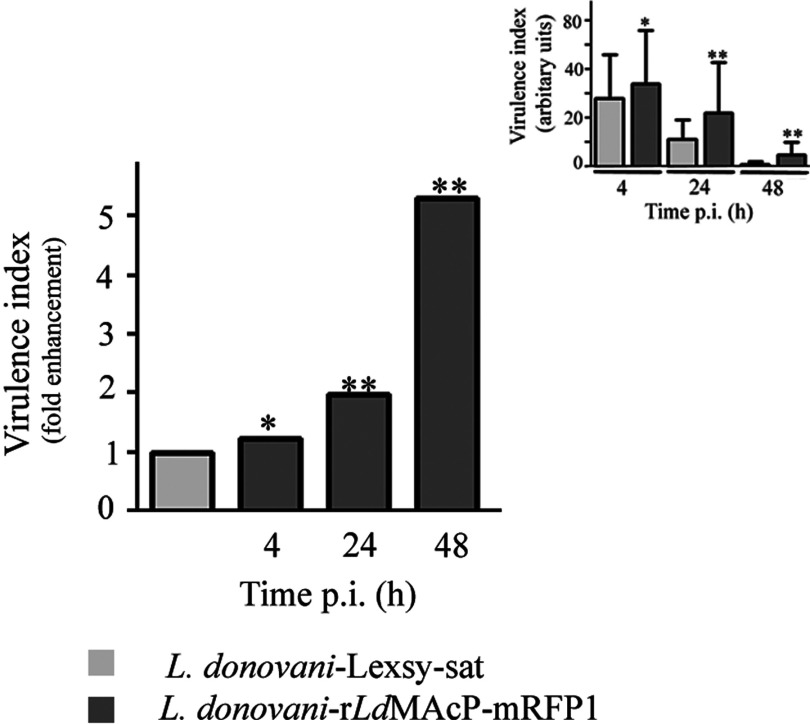
Infection of J774 macrophages with *L. donovani* r*Ld*MAcP–mRFP1 and *L. donovani* pLexsy-sat promastigotes: virulence index Infection of macrophages for 4, 24 and 48 h and evaluation of parasite virulence was performed as described in the ‘Experimental’ section. The virulence index (inset) was calculated by counting more than 500 macrophages per case in three independent experiments and is expressed as fold enhancement relative to the control values (*L. donovani* Lexsy-sat). Error bars are S.D.s from three independent experiments. *P<0.05 and **P<0.01 compared with corresponding control values using a two-tailed paired Student's *t* test.

In the first 4 h of infection, both transgenic parasitic populations infected equally ∼90% of the macrophages, whereas they presented no significant difference in the mean number of internalized parasites per macrophage (Supplementary Table S2). However, an initial difference detected 24 h post-infection (1.2-fold higher infectivity index and 2-fold higher virulence index) became more statistically significant 48 h post-infection with an approximately 2-fold higher infectivity index and ∼5-fold higher virulence index (Figure 6; Supplementary Figure S4). This difference could be due to a more efficient survival of the r*Ld*MAcP–mRFP1 overexpressing parasites inside the macrophages.

The efficiency of the parasite's survival in this infection system was further evaluated by a survival assay based on the fluorescent labelling with CMFDA of parasites recovered from the infected macrophages and further analysis by FACS (Experimental). The apoptotic/dead parasitic population was separated from the live population by appropriate gating (Supplementary Figure S5). This assay revealed at 72 h post-infection a small but consistent and statistically significant higher survival efficiency of the *L. donovani* r*Ld*MAcP–mRFP1 parasites as compared with the control parasites (5% ± 0.3%, mean value ± S.D. from three independent experiments, *P*<0.05, two-paired Student's *t*test).

Overall, these experiments indicate that overexpression of r*Ld*MAcP–mRFP1 improves the ability of the transgenic parasites to survive within macrophages in culture at least in the first 48–72 h of infection, suggesting a possible similar role for the endogenous *Ld*MAcP. This finding supports earlier reports which link the tartrate resistant acid phosphatase activity to *Leishmania* infectivity and virulence [18,19]. It is worth mentioning that the tartrate sensitive secreted acid phosphatase activity has also been linked to *Leishmania* infectivity [70,71].

It remains to be tested if the *Ld*MAcP enzyme also plays a role in the modulation of secretion of the macrophage's cytokines in the inflammatory response, which could affect the pathophysiology of the potentially lethal visceral leishmaniasis. The fact that *Ld*MAcP seems to be specifically expressed as ecto-phosphatase in the *L. donovani* complex, responsible for viscerilization, supports this hypothesis. The species specific sequence diversity of the *Ld*MAcP gene, as well as its strain polymorphism, which is expected to affect the localization and/or the enzymatic activity of the encoded protein, may be linked to disease symptoms and could be proven useful in diagnosis or epidemiological studies.

## CONCLUSIONS

Despite the fact that acid ecto-phosphatase activity was identified in *Leishmania* more than 30 years ago, the precise role of this activity in the parasite infectivity and virulence still remains to be elucidated. The possibility exists that other membrane bound acid phosphatases, besides *Ld*MAcP, the only acid ecto-phosphatase with known molecular identity thus far, may contribute to this activity. With the genomes of many *Leishmania* sp. entirely or partially sequenced, it is now possible to readdress open questions in this field. Overall, our work brings new insight into the field of *Leishmania* acid ecto-phosphatases, clarifies ambiguities related to the sequence, species specificity, localization and ecto-phosphatase activity of the *Ld*MAcP and will trigger new studies on the interesting family of *L. donovani* HAcPs and their role in parasite life and virulence.

## Online data

Supplementary data
