# Supplementary material for: The Leishmania donovani histidine acid ecto-phosphatase LdMAcP: insight into its structure and function
Source: Biochem J. 2015 Apr 17;467(Pt 3):473–86. doi: 10.1042/BJ20141371 (PMC4687092; doi:10.1042/BJ20141371)
Supplement: Supplementary data [file bj4670473ntsadd.pdf]

Figure S1. Amino acid sequence comparison of *Ld*MAcP orthologs in *Leishmania* spp.
ClustalW multiple sequence alignment of the *Ld*MAcP orthologs was applied on the following aa sequences: a) *L. donovani* (strain LG13) (AIF32067) generated in this work; b) *L. donovani* AcP-3.1 (tartrate resistant; AF149839); c) *L. donovani* AcP-3.2 (tartrate sensitive; AAG01046.1); c) *L. donovani* BPK282A1 (LdBPK_366740.1); d) *L. infantum* (LinJ.36.6740); e) *L. major* Friedlin (LmjF.36.6460); f) *L. mexicana* (LmxM.36.6480); g) *L. braziliensis* (LbrM.35.6820) and h) *L. tarentolae* Parrot-Tarll (LtaP34.3910). All sequences except the *Ld*MAcP-LG13, were obtained from EMBL and TriTrypDB databases. The output of ClustalW alignment was analyzed/ edited with the BioEdit Sequence Alignment Editor. Identical aa are boxed. Red vertical boxes indicate the sequence polymorphisms between the MAcP sequences from *L. donovani* strains, dark blue boxes the putative 23 aa SP (Met^{1}–Ala^{23}) (Signal 4.1) and the putative TM domain (Leu^{274}-Tyr^{302}) of *Ld*MAcP (TMHMM algorithm), green box the HAcP signature motif and the purple horizontal bar (Glu^{73}-Leu^{84}) the peptide sequence used to generate an anti-*Ld*MAcP monoclonal antibody. The sequence of *L. mexicana* (LmxM.36.6480) is presented up to aa 600.

Figure S2 Consensus tree derived from sequence alignment of *Ld*MAcP (AIF32067) and *Ld*SAcP1 (AAC79513) with several members of the HAcP superfamily of known tertiary structures.
Branch-2 harbors almost exclusively HAcPs and phytases, Branch 1 has much more diverse functions, being mostly phosphomutases. Capital letters stand for pdb codes of determined 3D structures. Proteins shown are the Human Prostatic Acid Phosphatase (1ND6), Human Prostatic Acid Phosphatase Precursor (2HPA), Rat Prostatic Acid Phosphatase (1RPA), E. coli Glucose 1-Phosphatase (1NT4), *E. coli* Phytase Acid Phosphatase (1DKQ), Human Erythrocyte 2,3 biphosphoglyceratemutase (1T8P), Human biphosphoglyceratemutase (2HHJ), Phosphoglyceratemutase homolog from *Bacillus stearothermophilus* (1EBB), Human Liver 6-Phosphofructo-2-Kinase/Fructose-2,6–biphosphatase (1K6M), *E. coli* Histidine Phosphatase SixA (1UJB), *A. fumigates* Phytase (1QWO). Branch numbers are indicated. The dendogram was generated by the use of the Phylip algorithm.

Figure S3 Detection of the r*Ld*MAcP-His protein expressed in BL21 *E.coli* cells with anti-*Ld*MAcP Abs
Total bacteria extracts from *E. coli* BL21 carrying the pTriEx1.1-*Ld*MAcP plasmid were analyzed by 12% SDS PAGE and immunoblotted with three different clones of anti-*Ld*MAcP mAbs 7B7, 17D5, 20C10 (lanes 1, 2, 3) (dilution 1:100), the anti-*Ld*MAcP mouse pAb (lane 5) (dilution 1:500) and the anti-His mouse mAb (lane 4) (1 μg/ml). Strips with the same quantity of *E. coli*-r*Ld*MAcP-His bacteria extracts were used. The Ab reactivity was revealed by the chromogenic DAB method. The arrow points to the r*Ld*MAcP-His band.

Figure S4 Infection of J774 macrophages with *L. donovani*-r*Ld*MAcP-mRFP1 and *L. donovani*-pLexsy-sat promastigotes. Infectivity index
Parasite infectivity was evaluated as described in Figure 6 and the Experimental section. Infectivity index (% No of infected macrophages/ total No of macrophages, Inset) is shown as fold enhancement of the control values (*L. donovani*-pLexsy-sat) for each time point. Error bars are Stds from 3 independent experiments. * $P < 0.05$ and ** $P < 0.01$ compared with corresponding control values (Lexsy-sat), using a two-tailed paired Student's t test.

Figure S5 Estimation by FACS of live and necrotic/apoptotic parasites from infected macrophages
J774 macrophages were infected with stationary phase *L. donovani* (strain LG13) promastigotes for 4 and 72 h. Parasites were released from macrophages by treatment with 0,01% (v/v) SDS, labeled with Cell Tracker Green (CMFDA) (5μM, 30 min, 37 ^{o}C) and analyzed by FACS. (**a**) CMFDA labeled *L. donovani* promastigotes; (**b**) CMFDA labeled necrotic/apoptotic parasites after 12 h treatment of parasite culture with 4 mM H_2O_2; (**c**, **d**)

CMFDA labeled parasites recovered from macrophages at 4 and 72 h p.i. M1: background FL1; M2: necrotic/apoptotic parasites; M3: live parasites.

Table S1. Primer sequences used in the PCR and qPCR reactions.

**Primer**	**Nucleic acid sequence**
**For mRFP1**	5'GAA GAT CTA TGG CCT CCT CCG AGG ACG 3'
**Rev mRFP1**	5'GGC CTC GAG TCA AGC TTC GAA TTC TTA GGC 3'
**For *Ld*MAcP (cloning in pTriEX1.1)**	5'GAA GAT CTC CAT GGC CTC GAA GCT CAT CCG TG 3'
**Rev *Ld*MAcP (cloning in pTriEX1.1)**	5' CCG CTC GAG ATA CAC GCG AAA TGC ATG AAG 3'
**For *Ld*MAcP (cloning in pLexsy-sat-mRFP1)**	5'GAA GAT CTC CAT GGC CTC GAA GCT CAT CCG TG 3'
**Rev *Ld*MAcP (cloning in pLexsy-sat-mRFP1)**	5' GAA GAT CTT CCT GAT CCT GAT GAT CC ATA CAC GCG AAA TGC ATG AAG 3'
**For *Ld*MAcPsol (cloning in pTriEX1.1)**	5' GAA GAT CTC CAT GGC CTC GAA GCT CAT CCG TG 3'
**Rev *Ld*MAcPsol (cloning in pTriEX1.1)**	5' CCG CTC GAG GAG GCG GTT GGC GTT GAT GTT G 3'
**For LmjF.36.6460**	5' GAA GAT CTC CCA AAT ACG GCA ACC ATG 3'
**Rev LmjF.36.6460**	5' CCG CTC GAG CAC AGG TTC ATC TAC AGG AC 3'
**For LtaP.3910**	5'GAAGATCT CC ATG ACC TCG AAG CTC ATC TGC 3'
**Rev LtaP34.3910**	5' CCG CTC GAG CAC GGT GTG TGG CAC AC 3'
**Rev LtaP.3910 (97 bp downstream the 3' end)**	5' CGTGTCGACAGCTGTCTCAC 3'
**For mRFP1 (qPCR)**	5' CTG TCC CCT CAG TTC CAG TA 3'
**Rev mRFP1 (qPCR)**	5' GGA AGG ACA GCT TCA AGT AGT 3'
**For *Ld*MAcP (qPCR)**	5' ATA GCT ACG TGT TGG TCT GC 3'
**Rev *Ld*MAcP (qPCR)**	5' ATA CAC GCG AAA TGC ATG AAG G 3'
**For GADPH (qPCR)**	5' GGG GCA CTT GTT GTG AAT G 3'
**Rev GADPH (qPCR)**	5' TGC TCC TGC TTC AAT GTG C 3'

Table S2. Parasite burden of J774 macrophages infected with *L. donovani*-r*Ld*MAcP-mRFP1 and control *L. donovani*-Lexsy-sat parasites.

Macrophages, inoculated with stationary phase promastigotes for 1 h at a ratio 1:20 parasites /macrophage were extensively washed to remove non internalized parasites and were examined for parasite burden by IF microscopy at 4, 24 and 48 h p.i. (Experimental). Results are mean values ± Stds from three independent experiments.

No of intracellular parasites per 100 macrophages		
Time p.i. (h)	*L. donovani*-Lexsy-sat	*L. donovani*-r*Ld*MAcP-mRFP1
4	144 ± 4.50	144 ± 3.50
24	142 ± 6.00	160 ± 4.00
48	131 ± 3.00	158 ± 5.70 *

Figure S1

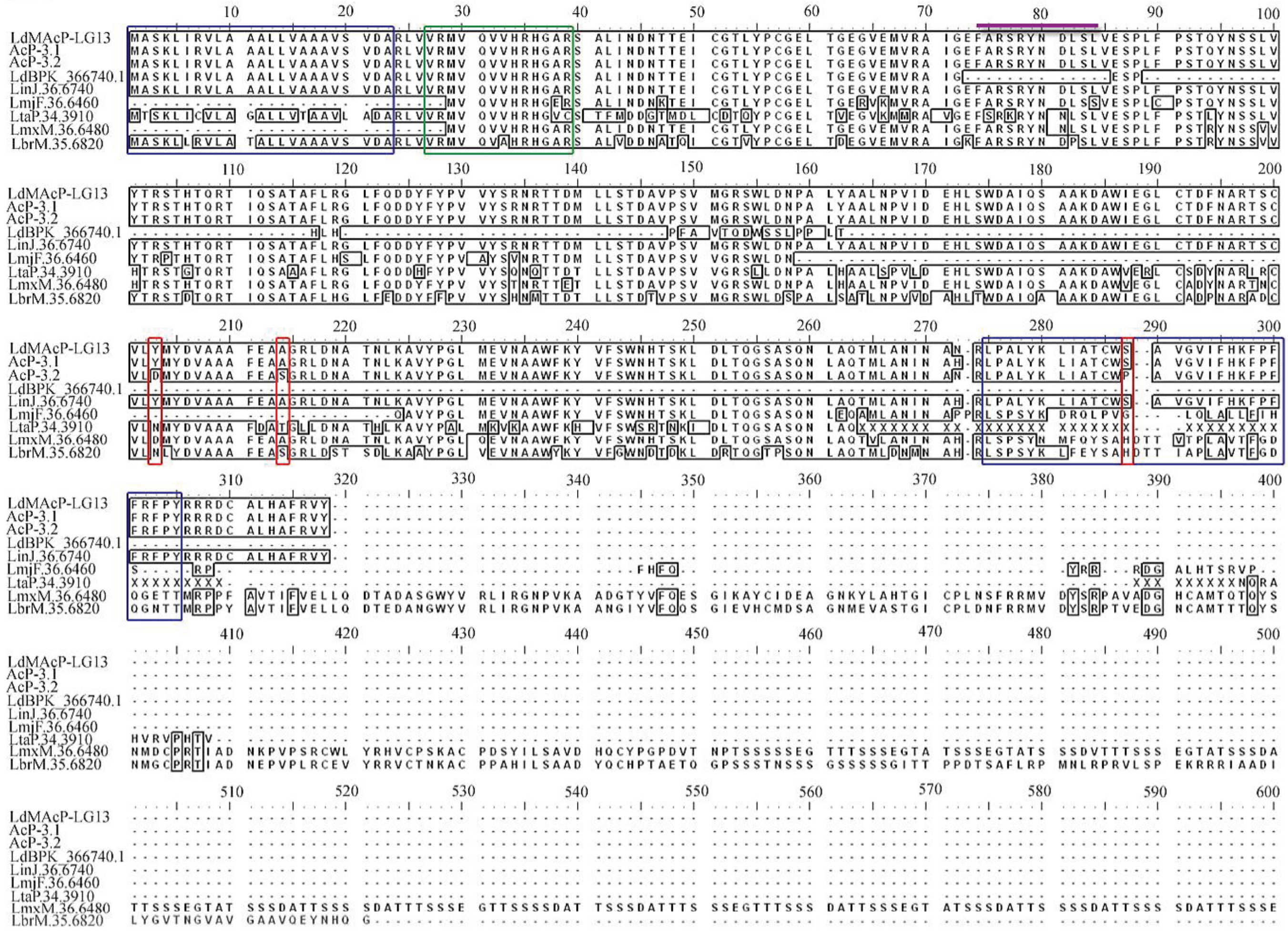
LdMAcP-LG13
AcP-3.1
AcP-3.2
LdBPK_366740.1
LinJ.36.6740
LmjF.36.6460
LtaP.34.3910
LmxM.36.6480
LbrM.35.6820

Figure S2

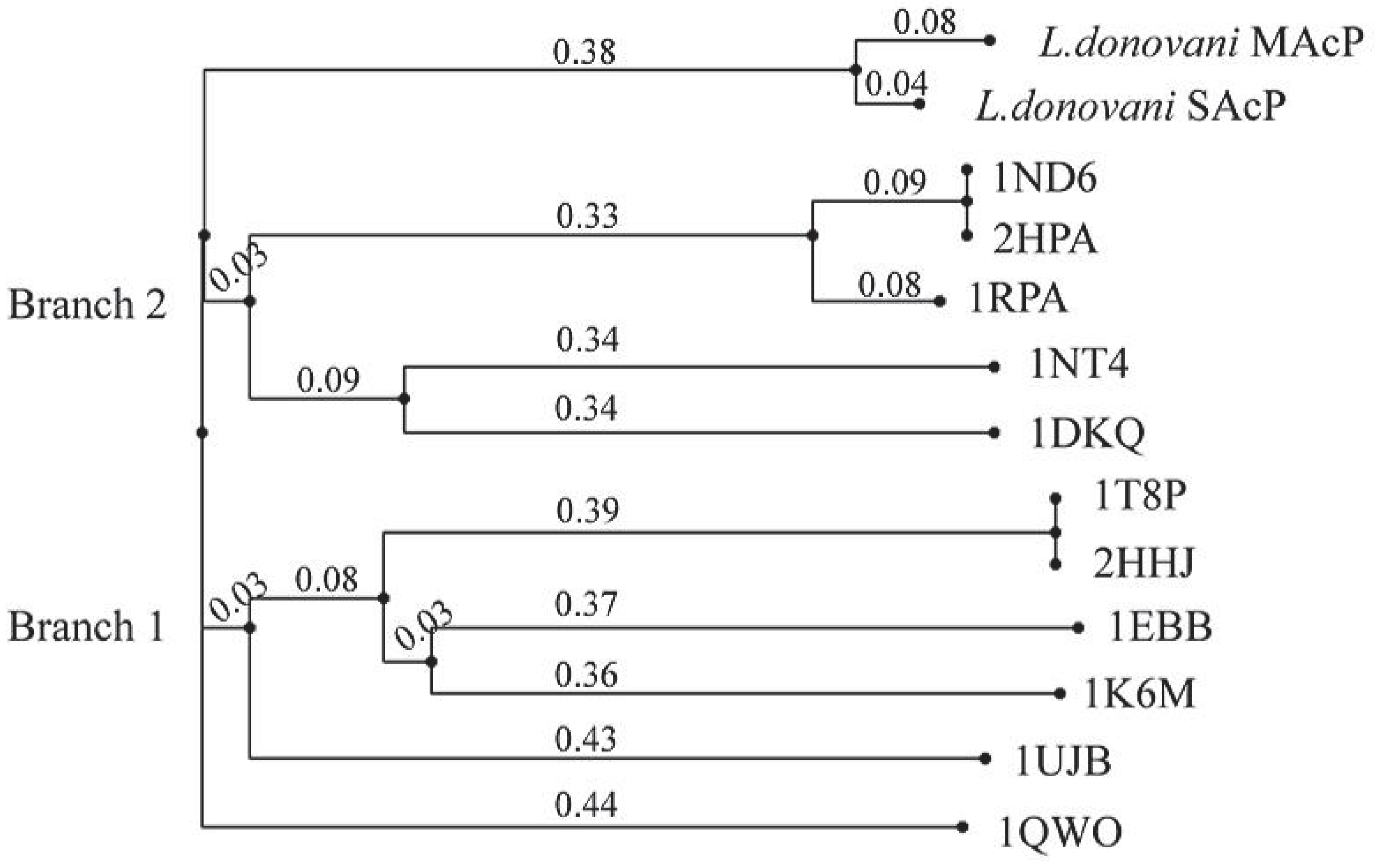
0.38
0.08
L.donovani MAcP
0.04
L.donovani SAcP
0.09
1ND6
0.33
2HPA
0.03
Branch 2
0.08
1RPA
0.34
1NT4
0.09
0.34
1DKQ
1T8P
0.39
2HHJ
0.03
0.08
Branch 1
0.37
1EBB
0.03
0.36
1K6M
0.43
1UJB
0.44
1QWO

Figure S3

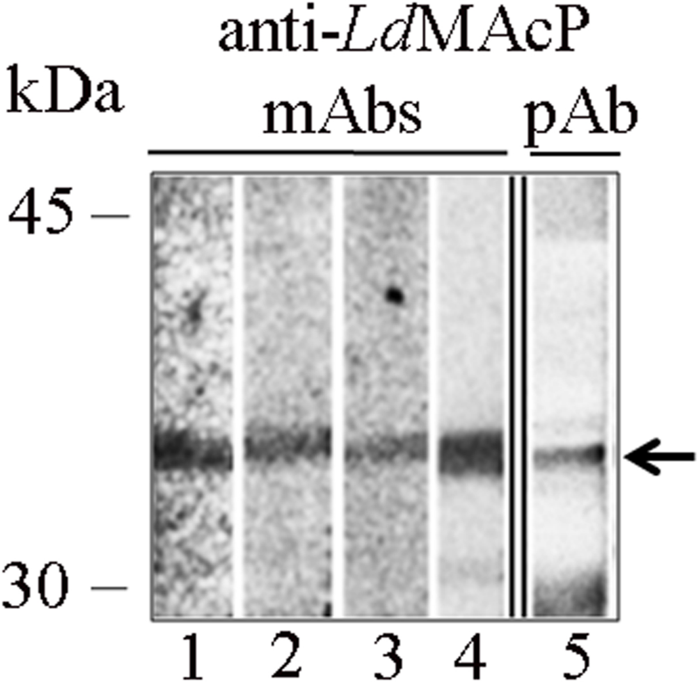

Figure S4

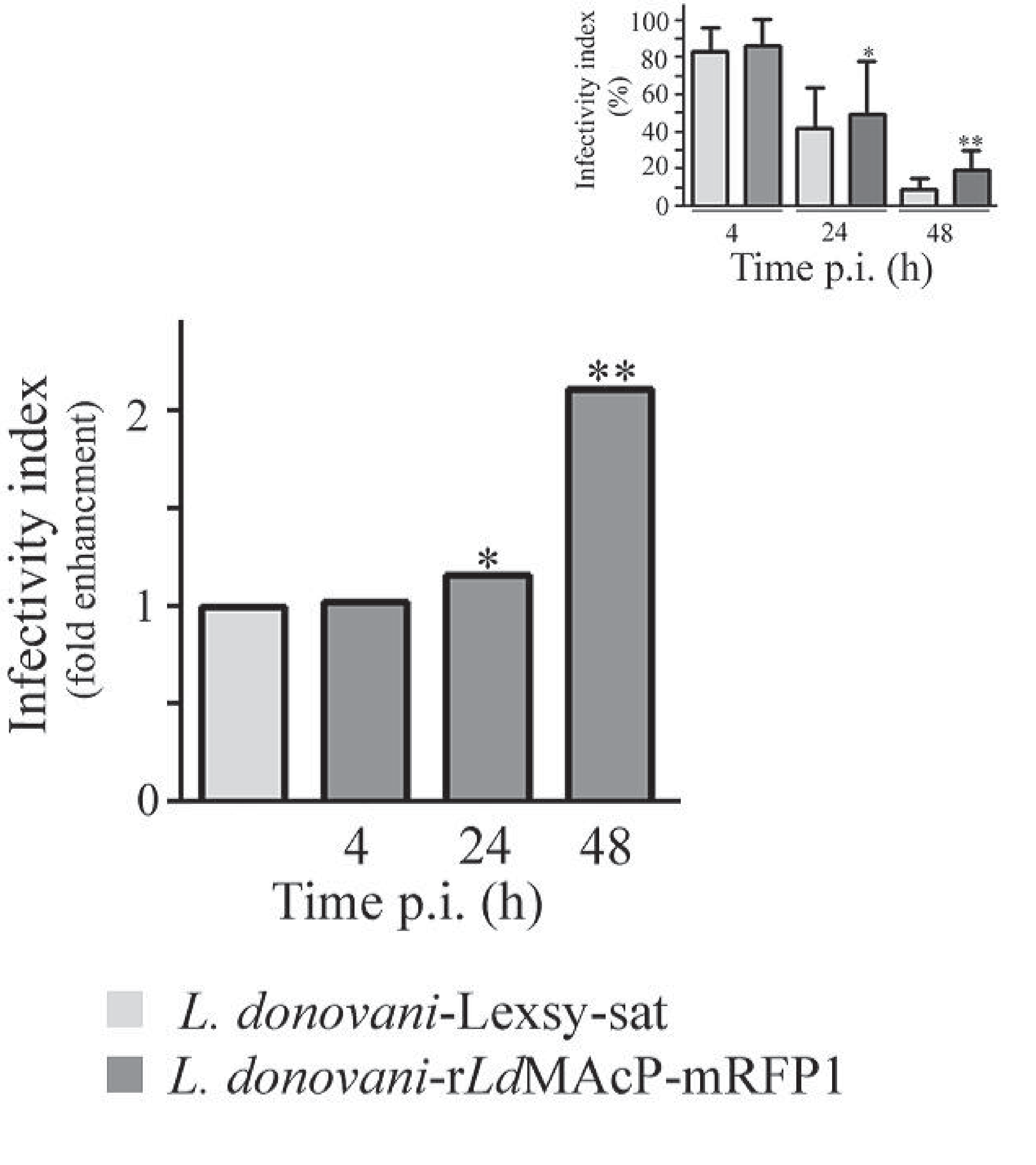
Infectivity index (%)
100
80
60
40
20
0
*
**
4
24
48
Time p.i. (h)
Infectivity index (fold enhancment)
2
1
0
*
**
4
24
48
Time p.i. (h)
L. donovani-Lexsy-sat
L. donovani-r*Ld*MAcP-mRFP1

Figure S5

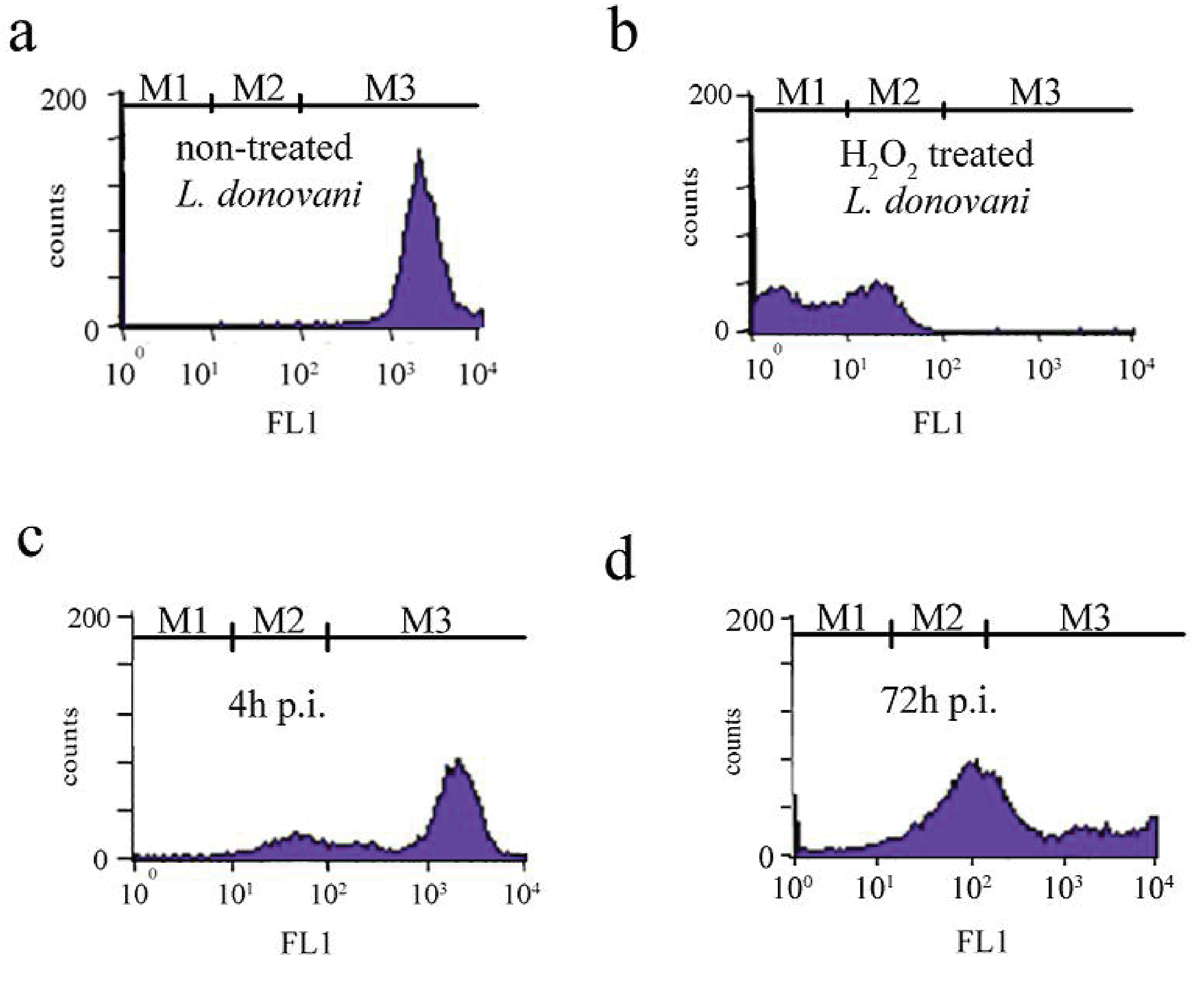
a
M1
M2
M3
200
0
counts
non-treated
L. donovani
10
10¹
10²
10³
10⁴
FL1
b
M1
M2
M3
200
0
counts
H_2O_2 treated
L. donovani
10
10¹
10²
10³
10⁴
FL1
c
M1
M2
M3
200
0
counts
4h p.i.
10
10¹
10²
10³
10⁴
FL1
d
M1
M2
M3
200
0
counts
72h p.i.
10⁰
10¹
10²
10³
10⁴
FL1
